# Integrating Social and Emotional Learning into Mathematics Education: A Multiple Case Study of JUMP Math’s Approach to Creating Socially and Emotionally Supportive Learning Environments

**DOI:** 10.3390/bs15101426

**Published:** 2025-10-20

**Authors:** Tonje M. Molyneux, Adele Diamond

**Affiliations:** 1 Educational and Counseling Psychology, and Special Education, Faculty of Education, The University of British Columbia, Vancouver, BC V6T 1Z4, Canada; 2 Developmental Cognitive Neuroscience Program, Department of Psychiatry, Faculty of Medicine, The University of British Columbia, Vancouver, BC V6T 2A1, Canada; adele.diamond@ubc.ca

**Keywords:** social and emotional learning, mathematics education, classroom climate, teacher well-being, student engagement, curriculum integration

## Abstract

Integrating social and emotional learning (SEL) into academic instruction may improve well-being and achievement. In mathematics—where anxiety and negative attitudes often hinder learning—SEL may be especially useful. This multiple case study examined how a math curriculum that explicitly embeds SEL principles shapes learning environments and teacher/student experiences. Using a multiple case study design, we conducted classroom observations, teacher interviews, and check-ins in six Grade 5–7 classrooms implementing JUMP Math, a program that centers social–emotional well-being. Three themes characterized the SEL-integrated environment: (1) Teaching Energy—steady pacing, enthusiastic delivery, and humor; (2) Learning Harmony—progressing together, peer help, and the normalization of mistakes; and (3) Emotional Stability—supportive feedback, invitations to participate, and respectful, responsive interactions. Teachers reported greater confidence and reduced math anxiety; students showed higher engagement, cooperation, and resilience in problem-solving. Findings indicate that math curricula intentionally designed with SEL can create emotionally supportive classrooms that benefit both teachers and students, while advancing academic goals. The findings contribute to understanding how academic instruction can be leveraged to develop social and emotional competence while maintaining focus on academic achievement.

## 1. Introduction

The recognition that social and emotional well-being is fundamental to academic success has led to increased interest in integrating social and emotional learning (SEL) into educational practice (Cipriano et al., 2023). Rather than treating SEL as a separate subject, researchers are exploring how social and emotional development can be fostered within academic instruction, creating synergistic effects that enhance both domains (Schwartz et al., 2023). This integrated approach is particularly relevant in mathematics education, where emotional factors such as anxiety (Barroso et al., 2021), doubts about self-efficacy (Holenstein et al., 2022), and peer relationships (Li et al., 2020) can significantly impede learning outcomes.

### 1.1. Social and Emotional Learning in Educational Contexts

Social and emotional learning encompasses addressing social and emotional needs as well as the processes through which individuals develop self-awareness, self-management, social awareness, relationship skills, and responsible decision-making competencies (Collaborative for Academic, Social, and Emotional Learning [CASEL], 2025; S. M. Jones et al., 2019). Meeting social-emotional needs and developing these competencies are not merely desirable additions to academic learning; they are fundamental to how students engage with educational content, interact with peers and teachers, and navigate the challenges of learning environments. Research consistently demonstrates that SEL programs positively impact academic performance as well as students’ social and emotional skills, attitudes, and prosocial behavior and reduce behavioral problems (Cipriano et al., 2023). For example, Cipriano and colleagues’ meta-analysis of universal school-based SEL programs found small to medium effect sizes for improvements to students’ social and emotional skills, positive social behaviour, academic performance, and attitudes toward self and others, and reductions in emotional distress and conduct problems. These benefits extend beyond immediate outcomes, contributing to long-term well-being, life satisfaction, and success in various life domains (Belfield et al., 2015; D. E. Jones et al., 2015; Moffitt et al., 2011).

The integration of SEL into academic curricula represents an evolution beyond standalone SEL programs. This approach recognizes that learning is inherently social and emotional (Zins & Elias, 2007), and that academic content can serve as a vehicle for developing social and emotional competence (Sears et al., 2022). When students engage in collaborative problem-solving, discuss their thinking processes, navigate challenges and setbacks, and support their peers’ learning, they are simultaneously developing academic knowledge and social-emotional skills (Martinez & Gomez, 2024). Contemporary educational contexts increasingly demand approaches that prepare students not only with academic knowledge but also with the social and emotional competencies necessary for success in an interconnected, rapidly changing world (Frey et al., 2019). The integration of SEL into academic instruction offers a promising pathway for addressing these dual needs while making efficient use of instructional time and creating coherent learning experiences for students.

### 1.2. Mathematics Education and Social-Emotional Factors

Mathematics learning is deeply intertwined with emotional and social factors, making it a particularly rich context for SEL integration. For example, math anxiety affects a significant proportion of students and teachers, creating barriers to engagement and achievement that can persist throughout individuals’ educational and professional lives (Ganley et al., 2019; Hembree, 1990; Luttenberger et al., 2018). This anxiety often stems from negative early experiences, competitive classroom environments, and beliefs about mathematical ability being fixed rather than malleable (Szczygieł & Pieronkiewicz, 2022). Beyond individual emotional responses, classroom social dynamics significantly influence mathematical learning. The quality of student-teacher relationships affects students’ willingness to take intellectual risks, persist through challenging problems, and engage actively in mathematical discourse (Crosnoe et al., 2010). Peer relationships shape students’ mathematical identities, their willingness to ask questions or admit confusion, and their access to collaborative learning opportunities that can deepen understanding (Francisco, 2013; Li et al., 2020).

Research has identified several emotional and social factors that support mathematics learning. Students perform better in mathematics when they experience positive classroom climates that evidence the following: (a) autonomy support: teachers provide choices, encourage initiative, and respect students’ perspectives; (b) authentic instruction: mathematical tasks connect to students’ lives and interests beyond school; (c) peer collaboration: opportunities to work together, share thinking, and learn from peers; and (d) teacher social support: warm, responsive relationships that communicate care and high expectations (Patrick et al., 2011; Wigfield et al., 2006). Additionally, classroom environments where mistakes are viewed as learning opportunities rather than failures support both mathematical understanding and social-emotional development (Dweck, 2006).

When students feel safe to make mistakes, ask questions, and engage in productive struggle, they can develop both mathematical resilience and broader life skills related to perseverance and growth mindset (Lee & Johnston-Wilder, 2017). These factors align closely with core SEL competencies, suggesting natural opportunities for integration. For example, self-awareness develops as students recognize their emotional responses to mathematical challenges and identify their strengths and areas for growth. Self-management emerges through persistence with difficult problems and regulation of anxiety or frustration. Social awareness grows through recognition of peers’ different approaches, styles, idiosyncrasies, and needs. Relationship skills develop through collaborative problem-solving and mathematical communication. Finally, responsible decision-making manifests in choosing appropriate strategies and considering the impact of one’s contributions on group work.

### 1.3. JUMP Math: A Curriculum Integrating SEL and Mathematics

JUMP Math, developed by Canadian mathematician Dr. John Mighton and staff at the Canadian charity JUMP Math, exemplifies an approach that explicitly integrates SEL principles into mathematics instruction. Founded on growth mindset principles (Dweck, 2006), the program aims to reduce math anxiety while building confidence, cooperation, and resilience alongside mathematical competence. The program’s philosophy centers on the belief that all students can achieve success in mathematics when provided with appropriate support, structure, and social-emotional scaffolding. The curriculum’s design reflects five key features that support both math learning and SEL development:

1. Structured Inquiry Approach: Concepts are broken down into smaller, manageable chunks that maintain students in what Mighton (2024) terms a “zone of productive struggle,” similar to Vygotsky’s zone of proximal development (Vygotsky, 1978). This approach reduces cognitive overwhelm while building confidence and self-efficacy. Students experience regular success while being appropriately challenged, fostering both mathematical understanding and emotional regulation skills.
2. Collaborative Learning Structure: The whole class works on the same concept simultaneously, with scaffolding adjusted to individual needs. This approach reduces comparison and fosters a supportive community of learners where peer assistance is normalized and valued. Students develop relationship skills through helping others and social awareness through recognizing diverse learning needs and approaches.
3. Mistake Normalization: The program encourages viewing errors as valuable learning opportunities rather than failures. Teachers and students regularly discuss mistakes, analyze their sources, and use them to deepen understanding. This practice develops resilience, reduces anxiety, and supports growth mindset development across both mathematical and social-emotional domains.
4. Reduced Comparison and Competition: By having all students work on the same problems with differentiated support rather than different levels of work, the program minimizes the social comparison that often undermines mathematical confidence and peer relationships. This structure supports positive classroom climate and inclusive learning environments.
5. Teacher Support and Development: Detailed lesson plans, professional development opportunities, and ongoing support help teachers develop both mathematical content knowledge and pedagogical skills. This support is particularly important given that many elementary teachers experience math anxiety themselves and may lack confidence in mathematical instruction. Thus, appropriate scaffolding is provided for both teachers and students.

Research demonstrates JUMP Math’s effectiveness for academic outcomes. A cluster randomized controlled trial found significant positive effects for elementary school math achievement (Solomon et al., 2019). Compared to students receiving traditional problem-based instruction, those in JUMP Math classrooms showed significantly greater progress in computation in Year 1 and problem-solving in Year 2. The National Book Fund’s yearly reports consistently show that math achievement in JUMP Math students grows at minimum 2.4 times the rate of standardized test norms (Murray, 2019). However, less is known about how JUMP Math’s SEL-integrated approach affects the broader learning environment, teacher experiences, and student social-emotional development. Understanding these impacts is crucial for comprehending the full potential of SEL-integrated academic instruction and identifying factors that support successful implementation.

### 1.4. Social and Emotional Learning and Teacher Well-Being

An important consideration in SEL integration is the role of teacher social and emotional competence and well-being (Schonert-Reichl, 2017). Teachers’ own social and emotional skills significantly influence their ability to create supportive learning environments, manage classroom dynamics effectively, and model positive social-emotional behaviors for students (Jennings & Greenberg, 2009). Jennings and Greenberg’s (2009) Prosocial Classroom Model suggests that teachers with higher social and emotional competence (SEC) are better able to create classroom climates that support both academic learning and social-emotional development. Further research has explored the relationship between educators’ SEC and pedagogical practice (Gimbert et al., 2021). For example, Hen and Goroshit (2016) found a significant and positive relationship between teachers’ emotional self-efficacy and teaching self-efficacy, suggesting that teachers who perceive themselves as being more social-emotionally capable also experience greater efficaciousness in their pedagogical practice.

Teacher math anxiety is particularly relevant in elementary education, where many teachers report feeling less confident and competent in mathematics than in other subject areas (Ganley et al., 2019; Ramirez et al., 2018). This anxiety can affect instructional practices, with anxious teachers spending less time on mathematics instruction, engaging in less mathematical discourse, and focusing more heavily on algorithmic procedures rather than on conceptual understanding (Bush, 1989; Karp, 1991; Trice & Ogden, 1986). Indeed, a large-scale replication study found that elementary students who learn math from highly math anxious teachers learn less math and have lower math achievement (Schaeffer et al., 2021). Curricula that support teacher confidence and competence may therefore have dual benefits: improving instructional quality and student math achievement while also supporting teacher well-being and professional development. When teachers feel more prepared and confident in their mathematical instruction, they may be better positioned to create the emotionally supportive environments that benefit student learning and development. Yet little research has explored how SEL-integrated math curricula can support teachers in these vital areas.

### 1.5. The Present Study

This study addresses a gap in understanding how a mathematics program explicitly designed with SEL principles creates socially and emotionally supportive learning environments and affects the experiences of both teachers and students. While research demonstrates the academic effectiveness of programs like JUMP Math, less attention has been paid to the social and emotional dimensions of their implementation and impact. Specifically, our study was guided by the following research questions: (1) What qualities characterize the learning environment during JUMP Math lessons? (2) How do teachers and students experience this approach to mathematical instruction? (3) What role does teacher implementation fidelity play in creating supportive learning environments? (4) How does SEL integration in JUMP math classrooms support the development of social and emotional competencies alongside academic achievement?

## 2. Materials and Methods

### 2.1. Research Design

This exploratory qualitative study employed a multiple case study design (Stake, 2013), enabling in-depth examination of teachers and students in authentic classroom settings while considering contextual dynamics that influence implementation and outcomes (Yin, 2009). The multiple case study approach facilitated both within-case analyses of individual classrooms and cross-case comparisons (Gustafsson, 2017; Yin, 2003, 2009) that enabled identification of patterns, variations, and factors associated with successful SEL integration. Case study methodology is particularly appropriate for investigating complex educational phenomena where context matters significantly and where the boundaries between the phenomenon of interest (SEL integration) and the context (specific classrooms, schools, and communities) are not clearly evident (Yin, 2003). This approach enabled a rich, nuanced understanding of how SEL integration manifests in real classroom settings during JUMP Math lessons, and how various factors influenced implementation and outcomes.

The first author conducted all classroom observations and teacher interviews and analyzed the data. She is a former classroom teacher, and current SEL program developer and educational researcher. The second author initiated and funded the study and supervised all research activities. She has conducted several classroom-based studies over many decades.

### 2.2. Setting and Participants

Data collection occurred in two public elementary schools (Kindergarten to Grade 7) in the lower mainland of British Columbia, Canada, during two time periods: Winter 2020 for School A (prior to COVID-19 lockdown) and Spring 2023 for School B. This timing allowed for examination of SEL integration across different contextual conditions while maintaining focus on the core phenomenon of interest.

#### 2.2.1. School Contexts

School A was located in a large urban district with higher population density (9246 people per km^2^) and median family income of $114,952, with 9.50% unemployment rate. School B was in a medium-sized district with lower population density (3607 people per km^2^) and higher median family income of $126,157, with 7.25% unemployment rate. For context, the 2020–2021 median family income in British Columbia was $98,460 and the unemployment rate was 8.9% (Statistics Canada, 2021a, 2021b), while Canada’s national median family income was $93,000 with an unemployment rate of 9.6% (Statistics Canada, 2021c). Both schools thus served communities with above-average incomes and, in the case of School B, below-average unemployment compared to provincial and national benchmarks. School A served 404 students in 2019/20, while School B served 610 students in 2022/23. Both schools served diverse student populations, with School A having majority white students and School B having majority students of color.

#### 2.2.2. Participants

Six elementary school teachers participated in the study. All were white female teachers with varying years of teaching experience (8–22 years) and JUMP Math implementation experience (1–15 years). Four teachers taught Grade 5 mathematics, one taught Grade 6, and one taught Grade 7. Mathematics was one of several subjects they taught, with all teaching in classrooms of fewer than 30 students. Teachers had diverse backgrounds in SEL training, ranging from no specific program experience to extensive training in multiple approaches. See Table 1 for complete case study demographic information.

The sample provided variation in both experience levels and contextual factors, enabling examination of how SEL integration manifests across different conditions while maintaining focus on the core phenomenon. All teachers were implementing JUMP Math as their primary mathematics curriculum, providing consistency in the SEL-integrated approach being examined.

### 2.3. Procedure

Ethics approval for this study was obtained from both the relevant school board review committee and university institutional review board. Teachers from various districts in the lower mainland of BC who were implementing JUMP Math were recruited through professional networks and district contacts. Following school district approval, teachers in participating schools were informed about the study through administrative channels and directly by researchers. Teachers participated voluntarily, with the clear understanding that the study focused on investigating learning environments rather than evaluating teaching effectiveness. Research activities included an introductory meeting to establish rapport and explain the study, semi-structured interviews to understand teacher backgrounds and perspectives, classroom observations during regular mathematics instruction, and check-in conversations to explore implementation experiences. Information about the study was provided to families of students in participating classrooms, who were given the option to opt out of classroom observations. No families chose to opt out. Researchers had no direct interaction with students during observations, maintaining focus on classroom dynamics and teacher-student interactions rather than individual student assessment or intervention.

### 2.4. Data Sources

Multiple data sources enabled triangulation and rich insights into how SEL integration manifested in mathematics classrooms, lending reliability and validity to findings through convergence of different types of evidence (Merriam, 2009).

### 2.5. Introductory Meeting and Semi-Structured Interviews

Prior to classroom observations, semi-structured individual interviews were conducted with each teacher to establish relationships and understand their backgrounds, perspectives, and experiences. Interviews explored teaching experience and perceived strengths, mathematics teaching background and any experiences of math anxiety, SEL training and implementation, and specific experiences with JUMP Math implementation.

Interview questions included:

1. How long have you been teaching, and what are your strengths as a teacher?
2. Some teachers experience anxiety around teaching math. Can you relate? Why or why not?
3. Do you think your students enjoy math? Feel successful at math? Why or why not?
4. Have you had training in SEL curricula like MindUP or Second Step?
5. How long have you been teaching JUMP Math? Was it your choice to implement it?
6. What kind of training did you receive in JUMP Math?

These questions enabled understanding of how teacher backgrounds, experiences, and perspectives might influence their implementation of SEL-integrated mathematics instruction.

### 2.6. Classroom Observations

Thirty-seven JUMP Math lessons were observed across the six classrooms, with 5–7 lessons observed per teacher. Observations were guided by a protocol based on the Classroom Assessment Scoring System (CLASS; Pianta et al., 2008), a widely used tool for assessing teacher-student interactions and classroom quality. For this qualitative research, CLASS domains, dimensions, and indicators provided an organizational structure for observational data rather than quantitative evaluation. Observations focused on three domains:

1. Emotional Supports: Including positive climate (relationships, affect, respect, communication), negative climate (punitiveness, sarcasm, disrespect), teacher sensitivity (awareness, responsiveness, addressing problems), and regard for student perspective (flexibility, autonomy, student expression)
2. Classroom Organization: Including behavior management (clear expectations, proactiveness, redirection), productivity (efficient time use, routines, transitions), and instructional learning formats (variety, engagement, clarity)
3. Instructional Supports: Including concept development (analysis, reasoning, creativity, integration), quality of feedback (feedback loops, encouraging responses, expanding performance), and language modeling (conversation, open-ended questions, mathematical language use)

This framework enabled systematic attention to both social-emotional and academic aspects of classroom interactions while maintaining flexibility to capture unexpected or unique aspects of SEL integration.

### 2.7. Check-In Conversations

Two follow-up conversations were conducted with most participating teachers during the observation period (COVID-19 restrictions prevented second conversations with two teachers). These exploratory conversations were designed to gain insight into teachers’ experiences implementing JUMP Math lessons and their perceptions of student experiences. Two open-ended questions guided these conversations: (1) Tell me about your experience teaching JUMP Math lessons; and (2) Tell me about your impression of your students’ experience during JUMP Math lessons. The non-leading nature of these questions allowed teachers to discuss anything they deemed relevant regarding their implementation experiences, student responses, challenges encountered, and benefits observed, providing rich data on how SEL integration was experienced by JUMP Math implementers.

### 2.8. Data Analysis

Data analysis comprised two phases following established qualitative research principles. First, individual case profiles were developed integrating data from interviews, observations, and conversations for each teacher. Second, cross-case comparisons identified patterns and variations across cases. Analysis followed the six phases of reflexive thematic analysis situated within a critical realist ontology (V. Braun & Clarke, 2022): (a) data familiarization through repeated reading and initial noting, (b) systematic open coding of interesting features across the dataset, (c) initial theme generation through collating codes into potential themes, (d) theme development and review through checking themes against codes and dataset, (e) theme refinement, definition, and naming to capture essential qualities, and (f) final analysis and writing that weaves together analytical narrative. This approach enabled identification of patterns while remaining sensitive to contextual variations and the complex, multilayered nature of SEL integration in mathematics education. The critical realist framework acknowledged that classroom phenomena exist independently of researchers’ perceptions while recognizing that understanding is constructed through interpretation of observable evidence.

## 3. Results

### 3.1. Overview of Themes

Across all six cases, three interconnected themes and related sub-themes were constructed that captured the qualities of the learning environment during SEL-integrated JUMP Math instruction. These themes reflected the multifaceted ways that SEL manifested within mathematical instruction, encompassing teacher behaviors, student interactions, and classroom climate characteristics. Evidence of these themes and sub-themes was found across all cases, although in some cases it manifested as absence or negative examples that illuminated the importance of these qualities for successful SEL integration. See Table 2 for a description of the themes, sub-themes, and examples.

The three themes that emerged from our observations align closely with JUMP Math’s five key design features described in the introduction. The Structured Inquiry Approach, with its emphasis on maintaining students in productive struggle zones, manifests primarily in Teaching Energy through steady instructional pacing that balances challenge with support, and in Emotional Stability through scaffolding that builds confidence. The Collaborative Learning Structure is evident in Learning Harmony, particularly in the sub-themes of progressing together and helping each other. Mistake Normalization directly corresponds to the Learning Harmony sub-theme of embracing mistakes. Reduced Comparison and Competition supports both Learning Harmony (through collective progress rather than individual achievement) and Emotional Stability (by creating psychological safety for risk-taking). Finally, Teacher Support and Development enables Teaching Energy by building teacher confidence for enthusiastic delivery and Emotional Stability by developing teachers’ capacity for responsive interactions. This alignment suggests that intentional curriculum design features can shape observable classroom practices and climate when implemented with fidelity.

### 3.2. Case Study Profiles: SEL Integration in Practice

The following detailed case profiles illustrate how SEL integration manifested differently across classrooms, demonstrating both successful implementation and challenges in creating socially and emotionally supportive mathematics learning environments.

#### 3.2.1. Case 1 Profile: Transforming Math Anxiety Through SEL-Integrated Instruction

The Case 1 teacher exemplified how SEL-integrated curriculum can support teacher well-being and competence while creating positive learning environments for students. With 20 years of teaching experience and 15 years of implementing JUMP Math, she served as both an experienced practitioner and mentor for other teachers. Her background included extensive training in social and emotional learning programs and restitution practices, providing a strong foundation for SEL integration. Significantly, this teacher had experienced intense math anxiety as a student, describing her struggles with mathematics in school as formative negative experiences that initially made her reluctant to teach the subject. However, she credited JUMP Math with transforming her relationship with mathematics, enabling her to overcome anxiety and gain confidence in mathematical instruction. This transformation was so profound that it inspired her to pursue graduate study in mathematics education, demonstrating the potential for SEL-integrated curricula to support not only student development but also teacher professional growth and well-being.

**Teaching Energy.** Case 1’s learning environment consistently featured *steady instructional pacing* that was responsive to learner needs rather than driven by curriculum coverage pressures. During one lesson where students needed to cut out triangles for a geometry activity, the teacher acknowledged the particular challenge this posed for students with fine motor difficulties. Rather than rushing the activity or applying pressure to finish quickly, she provided the time students needed while offering additional support and encouragement. This responsiveness demonstrated emotional awareness and social sensitivity—key SEL competencies—while ensuring that all students could participate meaningfully in the mathematical activity. The teacher’s *enthusiastic delivery* of content was evident in her explicit and rigorous use of mathematical language throughout lessons. She consistently used precise mathematical terms when reflecting students’ ideas and responses, modeling academic language while supporting students’ mathematical communication development. Students enthusiastically collected and defined mathematical terms in special notebooks, frequently encouraged to add new terms and review previously learned vocabulary. This practice created an environment that valued mathematical thinking and communication while building students’ confidence in using academic language. Students clearly felt at ease and comfortable in this mathematics learning environment, as demonstrated through their positive, friendly, light-hearted interactions that included natural *infusions of humor*. The teacher readily smiled and laughed with students, creating a relaxed atmosphere that likely helped reduce experiences of math anxiety for both herself and her students. This emotional climate supported risk-taking and engagement while building positive relationships that facilitated learning.

**Learning Harmony.** The mathematics learning environment in Case 1 showed abundant evidence of learning harmony through collaborative structures that supported both academic and social-emotional development. The teacher and students regularly *progressed together* through lessons at a common pace, with whole-class instruction serving as the predominant strategy. Lessons typically began with review of prior concepts and exploration of new material as a learning community, with opportunities for individual and partner work embedded within this collaborative structure. Importantly, the teacher consistently brought the class back together to reach common understanding of material, creating a sense of shared purpose and community rather than individual competition. This approach fostered learning and applying mathematical concepts cooperatively, building social awareness and relationship skills while supporting academic achievement. Cooperation was further evident in regular examples of students *helping each other* throughout lessons. During the triangle-cutting activity mentioned earlier, students who completed the task early naturally offered to help those who were struggling, without being directed to do so by the teacher. Other instances of students asking for or offering help occurred consistently across observations, with students regularly checking answers together and working through problems collaboratively. These interactions developed empathy, communication skills, and collaborative problem-solving abilities alongside mathematical understanding. Learning harmony was also promoted through the consistent practice of *embracing mistakes* as learning opportunities. The Case 1 teacher modeled this by acknowledging and learning from her own errors during instruction, celebrating student mistakes as chances for collective learning and understanding. This practice reduced anxiety, built resilience, and supported growth mindset development while creating a psychologically safe environment for mathematical risk-taking.

**Emotional Stability.** Emotional stability was intentionally fostered through the positive relationships the teacher built with her students, creating an environment where students felt acknowledged, respected, and accepted as mathematical learners. The teacher’s *supportive feedback* helped students feel encouraged and capable of taking intellectual risks, as evidenced by their willingness to propose unusual strategies and approaches to solving mathematical problems. Students clearly felt safe to think differently and explore alternative approaches rather than feeling pressured to arrive at one “right” answer through one “correct” method. This psychological safety supported creative mathematical thinking while developing self-confidence and autonomy in learning. The teacher demonstrated skill in *inviting participation* rather than demanding it, acknowledging that shifting emotional states can affect learning and being responsive to students’ varying needs and comfort levels. This approach aligned with the structured nature of JUMP Math lessons, which featured multiple ways to probe students’ thinking while scaffolding their learning incrementally. *Respectful and responsive interactions* were consistently observed between the teacher and students and among students themselves. The teacher acknowledged and respected students’ varying skill levels while empowering them to engage in ways that supported their individual learning needs. Students frequently checked answers with each other, worked through problems collaboratively, and offered encouragement when peers struggled with challenging concepts. These interactions developed social awareness, empathy, and communication skills while creating a supportive community of learners.

**Teacher Perspective on SEL Integration.** The Case 1 teacher’s experience illustrated the transformative potential of SEL-integrated mathematics curriculum for educators. Having struggled with math anxiety as a student, she found that JUMP Math’s structured approach and embedded teacher support significantly boosted her confidence and enjoyment in mathematics instruction. She was able to create a non-stressful, non-competitive environment grounded in the belief that all students can learn mathematics when provided with appropriate support and social-emotional scaffolding. The teacher particularly valued JUMP Math’s step-by-step approach and collaborative philosophy, which enabled both student success and natural peer mentoring opportunities. She noted some challenges with her school’s platooning approach, where students from different classrooms came together specifically for mathematics instruction, making it more difficult to build the same rapport and community bonds that facilitated SEL integration. However, even under these conditions, students demonstrated success and willingness to help each other. Remarkably, the teacher reported exceptional academic outcomes alongside social-emotional benefits: “Every student got between 90% and 100% on a division test!” She attributed this success to JUMP Math’s inclusive structure and its provision of entry points for all learners, including students with special needs. Specifically, she noted how the step-by-step approach supported a student with ADHD who had previously been labeled as defiant: “The step-by-step approach is helping her; she doesn’t get overwhelmed and shut down [now].”

#### 3.2.2. Case 2 Profile: Fostering Mathematical Discourse and Community

The Case 2 teacher brought 14 years of teaching experience and 8 years of JUMP Math implementation to her Grade 6 classroom, along with extensive background in social and emotional learning. She had received training in two specific SEL programs, completed coursework in mindfulness, and attended multiple seminars on social-emotional learning, providing her with a rich foundation for integration approaches. Interestingly, this teacher did not describe personal struggles with learning mathematics as a student. Instead, her math anxiety emerged during her teaching career when she realized she could not adequately explain underlying mathematical concepts to support student understanding. This recognition of her own learning needs demonstrated the self-awareness component of SEL and motivated her ongoing professional development in both mathematical content knowledge and pedagogical approaches.

**Teaching Energy.** The mathematics learning environment in Case 2 featured *steady instructional pacing* that the teacher engineered to be responsive, focused, and appropriately fast-paced to maintain student engagement and active participation. This pacing supported students’ ability to remain focused on mathematical concepts while feeling motivated to contribute their thinking and ideas to classroom discussions. *Enthusiastic delivery* was evident in the energetic questioning strategies the teacher used to help students extend and deepen their mathematical thinking. Students reciprocated this enthusiasm by frequently and excitedly offering their own ideas and connections about mathematical content, creating a dynamic learning environment characterized by high engagement and intellectual curiosity. The teacher consistently used specific, precise mathematical language throughout every observed lesson, continuously prompting and encouraging students to develop their own mathematical communication skills. Significant time and effort were devoted to building students’ mathematical vocabulary and discourse abilities. During one lesson where students demonstrated fluency with mathematical terminology, the Case 2 teacher remarked, “This is a great day!” She regularly called attention to mathematical thinking and its applications beyond school, encouraging students to consider how mathematics related to their everyday lives and the lives of others. Frequent *infusions of humor* helped create an energetic overall climate of enjoyment and enthusiasm about mathematical learning. The teacher used humor strategically to engage students and induce positive emotions related to mathematics, helping to reduce anxiety and build positive associations with the subject. Students often returned the teacher’s humor, creating an atmosphere of relaxed and friendly interaction that supported both relationship building and learning engagement.

**Learning Harmony.** Learning harmony was achieved through multiple structures and practices in the Case 2 classroom that supported both collaborative learning and social-emotional development. The classroom featured abundant examples of *progressing together* within a supportive learning environment that valued both mathematical thinking and community building. Similarly to Case 1, whole-class instruction was the primary format, with all students moving through mathematical content together at a common pace. However, this teacher skillfully created what she described as a “cooperative math learning community” where students felt comfortable taking intellectual risks, questioning concepts, making mistakes, and pushing their understanding through collaborative inquiry. During one observed lesson, students worked in small groups to identify and describe everything they could observe about a geometric shape (a rectangle divided into two equal parts with one diagonal line). Students engaged in lively mathematical discourse, with the teacher regularly bringing the whole class back together to learn from each other’s observations and ideas. The teacher reinforced mathematical language use and thinking while encouraging students to draw out and build upon each other’s insights.

Frequent examples of *helping each other* were evident throughout lessons, with students using questioning strategies to help peers clarify their thinking and explain their approaches to solving problems. The focus was on the process of developing and articulating mathematical reasoning rather than simply arriving at correct answers. This emphasis supported both mathematical understanding and communication skill development. Both teacher and students consistently *embraced mistakes* as valuable learning opportunities. The teacher readily acknowledged her own lack of understanding or misunderstanding during lessons, modeling the belief that abilities are not fixed and can be improved through effort and practice—a core growth mindset principle (Dweck, 2006). She accepted feedback from students and demonstrated how to learn from errors, creating a classroom culture where mistakes were viewed as natural parts of the learning process rather than failures.

**Emotional Stability.** The Case 2 mathematics learning environment successfully balanced high energy and enthusiasm with a steady, stable foundation of calm and focus that helped to create an emotionally safe place to learn math. This balance was crucial for supporting both academic risk-taking and social-emotional development. The teacher consistently modeled offering *supportive feedback* that helped students feel comfortable sharing unconventional or creative ideas. She validated out-of-the-box thinking and regularly invited the class to explore unusual approaches together, creating a learning community where intellectual risk-taking was valued and supported. This practice developed students’ confidence, creativity, and willingness to engage in mathematical reasoning. Students demonstrated comfort with collaborative problem-solving and learning from each other, indicating the development of relationship skills and social awareness. The teacher skillfully *invited participation* through varied instructional formats, including frequent partner and small-group activities embedded within whole-class instruction. This approach leveraged the social inclinations of Grade 6 students while supporting different comfort levels and learning preferences. *Respectful and responsive interactions* between the teacher and students, along with demonstrated enthusiasm for learning and abundant appropriate humor, fostered a positive learning environment where students felt acknowledged, accepted, encouraged, respected, and supported. Students exhibited these same qualities in their interactions with each other, indicating the development of empathy, social awareness, and relationship skills.

**Teacher Perspective on SEL Integration.** The Case 2 teacher had not experienced difficulty learning mathematics as a student but became aware of gaps in her own mathematical understanding when she attempted to explain concepts as a teacher. This realization motivated her ongoing professional development and demonstrated the self-awareness and self-management aspects of SEL. She approached her own learning with a growth mindset, which she actively promoted among her students. She particularly valued Grades 6 and 7 as pivotal years for addressing fixed mindsets about mathematical ability before students reached secondary school, emphasizing the importance of building mathematical confidence during these transitional years. The teacher identified several strengths of the JUMP Math curriculum that supported SEL integration, including its requirement that teachers develop deep understanding of mathematical concepts in order to support student learning effectively. She noted that “kids who have JUMP from Grade 1 are better mathematical thinkers,” suggesting that sustained experience with SEL-integrated mathematics instruction supports long-term development. She appreciated recent improvements to the curriculum that included more open-ended questions, providing appropriate challenge for students who needed it while maintaining step-by-step support for those who required more scaffolding. The teacher’s primary goal was for students to feel confident in mathematics, and she had observed “some who weren’t so confident step up,” which she attributed to JUMP Math’s design features that support both academic and social-emotional development. However, she noted that some students still preferred rote learning approaches and avoided deeper mathematical thinking, suggesting that changing established patterns requires sustained effort and support. The teacher highlighted benefits of her school’s platooning approach for mathematics instruction, including increased opportunities for socialization and engagement. She employed techniques like “My Favourite Mistake,” where students identified and discussed their errors with partners or the whole class, working collaboratively to understand the source of mistakes and correct them. This practice exemplified SEL integration by developing self-awareness, self-management, and relationship skills within mathematical contexts.

#### 3.2.3. Case 3 Profile: Building Grit and Determination Through Supportive Challenge

The Case 3 teacher brought 19 years of teaching experience to her Grade 7 classroom, though she was only in her second year of implementing JUMP Math. Her extensive background as a learning support teacher enhanced her empathy for students who struggled or needed additional patience and assistance, providing her with valuable skills for creating inclusive learning environments that supported diverse learners. Unlike some other case study teachers, she did not describe a personal history of mathematics anxiety either as a learner or educator. However, her considerable experience supporting struggling learners helped her understand the emotional and social dimensions of learning challenges. She expressed genuine enjoyment in teaching mathematics and felt adequately prepared through her JUMP Math training and ongoing support. While she had no experience with specific SEL programs, she had received training in the Heart-Mind approach and regularly used mindful breathing techniques with students to help reduce anxiety and support emotional regulation. These practices demonstrated her commitment to supporting students’ social-emotional well-being alongside academic achievement.

**Teaching Energy.** During classroom observations, multiple examples of *steady instructional pacing* were noted that supported both learning and emotional regulation. During one lesson reviewing polygon transformations in preparation for an upcoming assessment, the teacher guided the whole group through a series of focused practice activities that included questions for the whole class, independent work time, prompts and checks for understanding, and brief group debriefs. The steady rhythm established during this activity supported students’ ability to focus and attend to mathematical tasks while managing any anxiety about the upcoming assessment. The teacher’s *enthusiastic delivery* of JUMP Math lessons was evident through positive, energetic interactions with students as she probed their understanding and encouraged more thorough explanations of their mathematical thinking. She frequently asked students to show their work on individual whiteboards, responding energetically to their efforts and encouraging them to demonstrate perseverance and determination in their mathematical problem-solving. Her use of language consistently reinforced social-emotional learning goals alongside academic ones. She regularly used phrases like “Show me your grit and determination” and “I can see you working hard to figure this out,” connecting effort and persistence to mathematical success while building students’ self-awareness and self-management skills. Some evidence of *infusions of humor* were observed, particularly in one-on-one interactions with students who needed additional assistance. The teacher used appropriate humor to establish rapport with individual students while helping them overcome struggles and engage more fully in mathematical learning.

**Learning Harmony.** There was abundant evidence of learning harmony in the Case 3 classroom across all six observed lessons. Each lesson featured significant components of *progressing together* through mathematical concepts and activities, with students well-practiced at responding to questions in unison and displaying their work on whiteboards simultaneously. When working through problems as a whole group, students felt comfortable building on each other’s ideas and collaboratively stepping through problem-solving processes. Further evidence of *helping each other* was observed in every lesson, creating an atmosphere of cooperation and collaboration. Students sat in groups and regularly offered assistance to peers who were struggling with concepts or procedures. During one lesson, when a student had difficulty explaining the reasoning behind an answer, other students spontaneously offered guidance and alternative explanations, prompting the struggling student to reconsider the approach and ultimately arrive at the correct solution. This peer support developed both mathematical understanding and social-emotional competencies including empathy, communication skills, and collaborative problem-solving abilities. The classroom community was well-practiced in *embracing mistakes* rather than ignoring them or being discouraged by errors. This approach was consistently modeled by the Case 3 teacher, who did not dwell on student mistakes but rather used them as learning opportunities. She frequently encouraged students to persist by saying “Try again, [student name]” and regularly highlighted examples of her “favorite mistakes” during lessons, helping students understand how errors could illuminate important mathematical concepts or common misconceptions.

**Emotional Stability.** The supportive Case 3 mathematics learning environment was enhanced by consistent attention to promoting students’ emotional stability and well-being. The teacher encouraged emotional regulation and resilience through the *supportive feedback* she provided, including phrases like “Try, try again!” and “Hang in there for five more minutes. You’re building grit!” This language explicitly connected persistence and effort to both mathematical success and character development. The teacher demonstrated skill in *inviting participation*, particularly from students who were struggling or not attending well to lessons. With a consistently friendly, warm tone of voice, she used a variety of approaches to engage different students, such as “Come on, bud!” or “I want you doing this with me, [student name].” When she noticed students’ energy or attention waning, she proactively invited them to take a brief movement break—running a lap around the field—before returning ready to focus and apply themselves to mathematical work. This responsiveness demonstrated her recognition of the stamina and emotional regulation required for engaging with challenging mathematical concepts, and her willingness to support students’ diverse needs for maintaining engagement and focus. Such practices developed students’ self-awareness about their own learning needs and self-management strategies for maintaining attention and effort. The teacher consistently showed evidence of *respectful and responsive interactions* with students through her practice of circulating around the classroom during independent work time, checking in individually with students about their progress and understanding. She would ask questions like “How are we doing? Can I put the answer up?” to gauge students’ readiness and ensure they had adequate time to work through problems before moving to whole-class discussion. Students demonstrated similar respect in their interactions with each other. For example, when one student asked for additional thinking time before sharing ideas with a table group, other students readily acknowledged the request and waited patiently for their peer to be ready. This pattern of patience and respect added to the emotionally stable and supportive mathematics learning environment.

**Teacher Perspective on SEL Integration.** The Case 3 teacher described how she had intentionally changed her teaching approach to meet the diverse learning needs of her students rather than expecting all students to adapt to a single instructional method. This flexibility and responsiveness reflected key SEL competencies including social awareness and responsible decision-making in her professional practice. She observed significant changes in her students through JUMP Math implementation, noting that former struggling students were now helping others and asking insightful questions during mathematics lessons. This transformation suggested that the SEL-integrated approach was developing both mathematical confidence and social-emotional competencies such as empathy and communication skills. When students seemed disengaged from lessons, she increased rigor and challenge to stimulate motivation and interest, demonstrating her understanding of the connection between appropriate challenge and engagement. She commented that many students felt proud of their mathematical progress, with one-third of her students specifically mentioning mathematics in their personal reflections on growth and learning throughout the year. The teacher found the JUMP Math program provided sufficient challenge and depth that supplemental activities like mathematics contests were unnecessary. She felt confident that the curriculum was preparing her students well for secondary school mathematics, both academically and in terms of the confidence and problem-solving skills they would need for continued success.

#### 3.2.4. Case 4 Profile: Collective Success and Confidence Building

The Case 4 teacher brought a unique background to elementary mathematics instruction, having previously worked as a secondary school coach and careers teacher before transitioning to upper elementary education. As a mathematics learner, she described having “hated the subject” and perceived herself as “not good in math,” experiences that enhanced her empathy for students who struggled with mathematical concepts or took longer to grasp new ideas. However, as a teacher, she felt confident and fluent in mathematical content and instruction, demonstrating how negative early experiences need not determine later competence and confidence. Her first year implementing JUMP Math had included training through collaborative time with colleagues and district support, though her extensive background in coaching and student support provided valuable skills for creating positive learning environments. While she had no experience with specific SEL programs, she mentioned that “SEL issues” were consistently addressed in her classroom practice, suggesting an intuitive understanding of the importance of social-emotional factors in learning environments.

**Teaching Energy.** The energy in the Case 4 mathematics learning environment was consistently high during JUMP Math lessons, with most students eager to answer questions and engage in discussions about mathematical concepts they were learning. This enthusiastic atmosphere was facilitated by the vibrant energy the teacher infused into lessons, delivered with *steady instructional pacing* that made it easy for students to follow along and maintain engagement as a whole class. At times, excitement about particular learning topics or student accomplishments caused the energy and pace to increase, creating moments of heightened engagement and celebration. However, for some of the quieter, more reserved students, these periods of intensified energy and quickened pace seemed somewhat overwhelming, highlighting the importance of balancing enthusiasm with sensitivity to diverse comfort levels and learning preferences. Similarly, while the teacher’s incredibly *enthusiastic delivery* of JUMP Math lessons was embraced and appreciated by most students, it occasionally felt daunting for students who preferred quieter, more reflective learning environments. During one lesson where the teacher invited students to demonstrate their methods for doubling two-digit numbers at the board, nearly every hand in the classroom shot up to volunteer. When the teacher called on a student who had not volunteered, other students groaned in disappointment at not being selected, while the chosen student declined to participate, likely feeling anxious about the public attention. The teacher respected the student’s choice and selected another volunteer but continued to encourage quiet students to participate verbally in validating solutions, sometimes requiring significant coaxing that could feel somewhat coercive. This highlighted the complexity of balancing encouragement with respect for individual comfort levels and the importance of developing sensitivity to diverse social-emotional needs. Despite these challenges, frequent *infusions of humor* brought levity and warmth to classroom dynamics. Lessons were filled with good-natured joking between teacher and students about “silly mistakes” and celebrating “math whizzes,” creating a light-hearted atmosphere that reduced anxiety and made mathematics learning feel welcoming and enjoyable for most students.

**Learning Harmony.** Students in the Case 4 classroom experienced a mathematics learning environment characterized by strong learning harmony and collaborative support. The classroom was arranged in a U-shape with all students facing the same direction, facilitating whole-class instruction and shared focus on mathematical concepts and problem-solving processes. The majority of lesson time involved *progressing together* through problems, with some time devoted to individual students demonstrating solutions at the board while others worked through the same problems on individual whiteboards. The teacher consistently waited for all students to achieve correct answers before moving on to new problems or concepts, ensuring that the learning community advanced together rather than leaving some students behind. Students who grasped concepts quickly were invited to try more challenging variations, such as multiplying three-digit numbers instead of two-digit ones, providing appropriate extension while maintaining the collaborative structure. This approach supported diverse learning needs while preserving the sense of collective progress and shared success. Learning harmony was further enhanced through consistent practices of *helping each other* throughout lessons. Students who were grasping concepts more slowly received hints and encouragement from peers, with the teacher actively promoting the idea of collective success and community learning rather than individual competition or achievement. During one lesson when an introverted student hesitated to answer a multiplication problem, the teacher said, “[Student name] knows the answer, she’s just working on her confidence,” while other students nodded understandingly and waited patiently, whispering words of encouragement. Struggling and making mistakes were normalized within this supportive community structure. The teacher modeled *embracing mistakes* by deliberately making errors for students to catch and correct, turning potential sources of embarrassment into opportunities for collaborative problem-solving and learning. Students readily admitted when they made mistakes and adopted the class custom of calling them “silly mistakes” that could be easily corrected through collective effort.

The teacher used questioning strategies to help students identify their errors and modeled the use of mathematical language when discussing mistakes and correction processes. These practices helped create a psychologically safe and supportive mathematics learning environment where intellectual risk-taking was encouraged and supported.

**Emotional Stability.** Emotional stability in the Case 4 classroom was promoted through consistently friendly, casual, positive interactions between the teacher and students and among students themselves. The teacher provided extensive *supportive feedback*, including inviting the class to give “a little ripple” (small clap) when someone grasped a concept or arrived at a correct solution, celebrating both individual achievement and collective success. Students had clearly become accustomed to the teacher frequently *inviting participation* in various forms, such as demonstrating solutions at the board, because nearly every student eagerly requested to be selected for these opportunities. The teacher also used alternative participation strategies that included everyone, such as asking for thumbs-up signals to show agreement or understanding, ensuring that all students could contribute regardless of their comfort with public speaking or demonstration. *Respectful and responsive interactions* contributed significantly to the emotional stability of the classroom environment. Students cheered for each other when individuals demonstrated solutions at the board and remained quiet when peers needed time to think through problems. The teacher normalized students’ struggles with challenging concepts like the long division algorithm, responding with warmth and encouragement when students became frustrated or impatient with their progress. When students became overly discouraged or overwhelmed, the teacher invited them to step away from the problem temporarily and return to it when they felt ready, demonstrating respect for individual emotional needs while maintaining high expectations for learning and persistence.

**Teacher Perspective on SEL Integration.** The Case 4 teacher’s initial skepticism about JUMP Math had been overcome through positive feedback from colleagues and her direct experience with the program’s benefits. She particularly appreciated that lessons were “all laid out for you [so you] don’t need to scramble,” valuing the structured support that reduced her planning burden while increasing her confidence in mathematics instruction. She was impressed by the structured lessons and her ability to adjust pacing to maintain student engagement and motivation. Most significantly, she observed that her students were having “’Yes!’ moments of success” with notably high motivation and engagement, evidenced by their requests to solve additional problems at the end of lessons and their voluntary mathematical work on whiteboards during free time. Even students who had previously struggled with mathematics were experiencing success, with recent assessment scores showing all students achieving 22 out of 30 points or higher. This improvement in both confidence and achievement suggested that the SEL-integrated approach was supporting both academic and social-emotional development simultaneously.

#### 3.2.5. Case 5 Profile: When SEL Integration Breaks Down

Case 5 provided crucial insights into how limited teacher social and emotional competence can undermine even well-designed SEL-integrated curricula. The teacher was in only her second year implementing JUMP Math and had received some training through collaborative time with colleagues, though the extent and quality of this preparation appeared insufficient for successful implementation. While she did not have specific training or experience with SEL programs, she mentioned employing some practices based on a school-wide approach to SEL and well-being. However, the implementation challenges observed in this classroom highlighted the critical importance of adequate preparation and ongoing support for teachers implementing SEL-integrated approaches. The classroom environment presented significant challenges, including a cramped and crowded physical space that often accommodated students from other classes who could not participate in swimming lessons with their own teachers. These contextual factors created additional stress and complexity that may have contributed to implementation difficulties.

**Teaching Energy.** In contrast to the other case study classrooms, *steady instructional pacing* was not consistently evident in Case 5. Instead, JUMP Math lessons were delivered with a staccato rhythm characterized by frequent interruptions from off-task students and the teacher’s attempts to manage challenging behaviors. This disrupted pacing created confusion and stress rather than the calm, focused learning environment that supported both academic and social-emotional development. There appeared to be poor fidelity to JUMP Math’s intended approach to teaching and learning. Due to the ongoing need to address behavioral issues, lessons were often hurried or incomplete, with the teacher skipping sections of lessons and jumping around the lesson plan in ways that compromised both mathematical understanding and community building. This fragmented delivery style contrasted sharply with the enthusiastic, engaging instruction observed in other classrooms. Instead, the teacher conveyed urgency to cover material, which resulted in students missing key mathematical concepts and feeling unsuccessful rather than supported and challenged. When students attempted to provide input about how to approach mathematical problems, the teacher emphasized that she was the one responsible for teaching, shutting down the collaborative discourse that characterizes effective SEL integration.

The teacher’s attempts at infusions of humor more closely resembled what Massey (2021) terms “snark”—a type of verbal aggression that uses sarcasm to diminish recipients rather than building positive relationships. An example of this inappropriate humor occurred when students tried to help each other with a division problem and the teacher responded sarcastically: “I know you like to help each other, but then what am I doing here?” Students did not respond positively to these interactions and appeared deflated rather than encouraged. The negative impact of this approach was evident when one student was overheard saying, “There’s no meaning of this in life,” during a division lesson in which most students were confused and feeling unsuccessful. This comment suggested that the lack of supportive *teaching energy* was undermining not only mathematical learning but also students’ sense of purpose and engagement in education more broadly.

**Learning Harmony**. The Case 5 classroom was physically arranged to support individual work, with students’ desks spaced apart in irregular rows rather than the collaborative groupings observed in other classrooms. While the teacher attempted to have students *progressing together* through lessons, frequent interruptions and behavioral issues consistently diverted attention from mathematical content and community building. The teacher tried to redirect students’ attention with comments like, “I haven’t seen your board in a while. Are you actually doing math?” and “Are you doing math right now? No, you’re drawing pictures.” However, these redirections were often ineffective and contributed to a negative classroom climate rather than supporting engagement and learning. Several students were consistently disengaged from instruction, only participating when extension questions offered more challenging material. These same students sometimes resisted the repetitive, step-by-step quality of JUMP Math lessons, to which the teacher responded: “I know this is tedious and you don’t enjoy going step-by-step, but for your classmates who need it, let’s keep at it.” While this response acknowledged different learning needs, it framed the curriculum negatively rather than helping students understand its benefits for building understanding and confidence. Although students were occasionally observed *helping each other*, this collaborative behavior was largely discouraged by the teacher, who viewed peer interaction primarily as disruption rather than valuable learning opportunity. Side conversations, whether about mathematics or other topics, were strongly discouraged with threats such as “I’m going to kick you out of the classroom if you don’t stop talking.” Students were primarily confined to individual work on whiteboards with their answers checked by the teacher, eliminating the collaborative learning structures that support both academic and social-emotional development. *Embracing mistakes* was not evident in this classroom environment. Both students and teacher expressed frustration when students struggled with concepts or failed to arrive at correct solutions quickly, creating anxiety and discouragement rather than resilience and growth mindset.

**Emotional Stability.** The Case 5 classroom was not characterized by emotional safety or stability. Students frequently appeared agitated, and the overall atmosphere was tense and confrontational rather than supportive and encouraging. Little *supportive feedback* was observed from teacher to students or among students themselves. Instead of *inviting participation* in ways that respected individual comfort levels and built confidence, the Case 5 teacher often demanded compliance and issued threats when students did not meet her expectations. Comments such as “If you’re not on task, then I will kick you out of the class and I don’t care if you cry or anything” created fear and anxiety rather than motivation and engagement. *Respectful and responsive interactions* were not evident in teacher-student or student-student relationships. Students sometimes picked on each other, shaming peers for mistakes and engaging in arguments unrelated to mathematics. The teacher struggled to respond effectively to students’ diverse social-emotional needs, often reacting to challenging behaviors with sarcasm, threats, and coercion that proved counterproductive. Some students responded to this treatment by standing up for themselves, with one student stating forcefully, “Stop calling me out” when repeatedly targeted by the teacher. While this response showed some resilience, the overall emotional climate was not conducive to effective teaching, learning, or social-emotional development.

**Teacher Perspective on SEL Integration.** Despite the significant implementation challenges observed in her classroom, the Case 5 teacher recognized some of the intended benefits of JUMP Math’s design. She appreciated that the program fostered deeper understanding of mathematical concepts, commenting that “It helps them learn the ‘why’ and makes them think” rather than simply memorizing procedures. She valued the scripted, well-structured layout of lessons and the breakdown of complex concepts into smaller, manageable steps. However, she found the amount of content challenging to cover within the academic year, noting that the content-rich JUMP Math lessons took her two and a half days to complete rather than the intended single day, leading to concerns about not completing the Grade 5 program by year’s end. She described ongoing challenges with inattentive students and chronic absenteeism that hindered student progress and made it difficult to build the consistent classroom community essential for effective math learning and SEL integration. While some students initially resisted new problem-solving strategies due to prior learning with different methods, she appreciated JUMP Math’s introduction of multiple approaches and the opportunity to offer students choices in their mathematical work. The Case 5 teacher’s experience illustrated how contextual challenges, insufficient preparation, and limited social-emotional competence can undermine even well-designed curricula, highlighting the crucial importance of adequate support systems for successful SEL integration.

#### 3.2.6. Case 6 Profile: Joy and Mathematical Community

The Case 6 teacher brought strong mathematical background and genuine enthusiasm to her first year of JUMP Math implementation. Having completed courses in calculus and obtained a Master of Business Administration, she possessed solid mathematical content knowledge that supported her confidence in instruction. Her expressed love for mathematics teaching and learning created optimal conditions for demonstrating the potential of SEL-integrated mathematics education. Despite being new to JUMP Math, she had received training from district administrators and collaborative time with teaching colleagues, though her mathematical background and teaching experience provided a strong foundation for implementation. Her approach to SEL included practices such as mindful moments, breathing techniques, and intentional community building, though she had not received training in specific SEL programs.

**Teaching Energy.** The Case 6 teacher clearly relished teaching JUMP Math lessons to her Grade 5 students, and her enthusiasm was met with focused, engaged learning from students. *Steady instructional pacing* was evident across all six observed lessons, with a consistent, unhurried rhythm that allowed students time to think and process concepts, particularly when working with complex topics such as division algorithms and fraction concepts. The teacher’s *enthusiastic delivery* reflected genuine interest in the foundational mathematical concepts underlying lesson topics, and she regularly took time to probe students’ thinking about different aspects of problems and procedures. One particularly engaging discussion centered on the difference between the fractions 9/2 and 8/2, with the teacher’s enthusiasm for deeper exploration resonating with students and prompting increased engagement with mathematical reasoning. Her genuine curiosity about mathematical thinking and willingness to explore concepts thoroughly created a classroom culture where intellectual curiosity was valued and mathematical exploration was enjoyable rather than stressful or competitive. *Infusions of humor* were abundantly evident throughout lessons in the Case 6 classroom, with the class regularly taking time to laugh together about mathematical situations and concepts. When introducing the concept of remainders in division, students and teacher laughed together about the scenario of dividing strawberries equally between two people when they didn’t have a knife and weren’t allowed to bite the fruit.

These shared moments of delight and laughter infused a light-hearted quality into the mathematics learning environment that reduced anxiety, built positive relationships, and created positive associations with mathematical learning. The humor was always appropriate and connected to mathematical content rather than being used to manage behavior or deflect from learning challenges.

**Learning Harmony.** The Case 6 classroom evidenced harmonious mathematics learning environment across all observed lessons, with students well-accustomed to *moving together* through JUMP Math activities using established routines and procedures. Students had developed effective collaborative learning habits including starting lessons with mental mathematics practice, solving problems on erasable boards, saying answers aloud together, and using non-verbal responses during checks for understanding. The teacher skillfully used questioning strategies to engage all students in exploring mathematical concepts and explaining their own thinking or helping to understand others’ approaches. This practice developed both mathematical communication skills and social-emotional competencies such as empathy and perspective-taking. Students were regularly observed *helping each other* throughout lessons, with this collaborative support actively encouraged by the teacher. When a student demonstrating a solution at the board encountered difficulties, the teacher would ask, “Who can help?” and multiple students would offer assistance and alternative explanations. During one lesson about measurement, students needed to use rulers and tape measures to assess various objects in the classroom. When some students struggled with proper tool use, others naturally offered help without being prompted. After one particularly successful lesson, a student complimented the teacher by saying, “You did great!” then corrected himself to say, “*We* did great!” This interaction demonstrated that students understood themselves to be part of a collaborative learning community where success was shared rather than individual, reflecting the development of social awareness and relationship skills alongside mathematical competence. The Case 6 classroom demonstrated consistent practice of *embracing mistakes* as valuable learning opportunities. When a student made an error by skipping steps while estimating with rounding, the teacher responded positively: “I’m really glad you did that. A lot of kids do that [mistake],” before guiding the whole class through the correct procedure. This response normalized the error while providing learning for all students. In another lesson, the teacher deliberately solved several division problems incorrectly on the board and asked students to help identify and fix her mistakes. She said, “Did I tell you I was a super genius? Even super geniuses make mistakes!” Students delighted in helping correct the errors while the teacher supported them in using precise mathematical language to explain their reasoning and solutions.

**Emotional Stability**. The emotional climate of the mathematics learning environment in Case 6 was notably stable and supportive, enabling both academic risk-taking and social-emotional growth. Students were comfortable with established routines and knew what to expect in lessons, creating a predictable foundation that supported their willingness to take intellectual risks and try new approaches to deepen their mathematical understanding. The teacher provided abundant *supportive feedback* ranging from physical encouragement (pats on the back) to frequent verbal affirmation with comments such as “Good job!” She also offered reassuring support when concepts were challenging, saying things like “It was a little bit of a tricky one, so don’t worry if you don’t get that one. In Grade 5, we work with whole cookies, not decimals.”

Students provided supportive feedback to each other as well, celebrating peers’ efforts to use estimation strategies during whole-class problem-solving activities and offering encouragement when classmates struggled with difficult concepts. The positive classroom climate was further enhanced by the teacher’s skill in *inviting participation* in ways that respected individual comfort levels and built confidence over time. She circulated throughout the classroom to check individual students’ understanding and invited some to demonstrate solutions at the board, making efforts to include reluctant students in supportive ways. When one student decided not to demonstrate at the board, the teacher did not pressure or dwell on this choice. When another student agreed to demonstrate but requested to bring a friend for moral support, the teacher readily accommodated this need, showing flexibility and responsiveness to individual social-emotional needs. *Respectful and responsive interactions* were commonplace throughout the Case 6 classroom, creating what observers described as an excellent mathematics learning environment. Interestingly, when a substitute teacher delivered a JUMP Math lesson to these students, the established routines and lesson structure supported some continued learning, but the lack of respectful and responsive behavior from the substitute clearly frustrated students, some of whom laid their heads down in discouragement. This contrast highlighted how crucial teacher social-emotional competence is for creating positive learning environments, even when curricula are well-designed.

**Teacher Perspective on SEL Integration**. The Case 6 teacher observed that her students were experiencing greater success learning mathematics with JUMP Math despite her concerns that they were making slower progress through the provincial Grade 5 mathematics curriculum compared to students in previous years. This tension between depth and coverage reflected common challenges in implementing SEL-integrated approaches that prioritize understanding and community building alongside content mastery. While she expressed some concern about the pace of curriculum coverage and her students’ preparation for Grade 6, she also recognized significant benefits in terms of student confidence and engagement. She felt that this year’s students “avidly embraced the challenge” of JUMP Math lessons and were “becoming confident in themselves as math learners” in ways that she had not observed in previous years with different curricula. Initially, she had worried that JUMP Math lessons were overly teacher-focused and might not provide sufficient opportunities for student agency and participation. However, she observed that her current students were actually participating more actively in mathematics classes and demonstrating greater excitement about mathematical learning than students in past years who had used different programs. She noted specific improvements in mathematical understanding, commenting that all of her current students felt confident with base-10 concepts, whereas in previous years this had consistently been a challenging area for many learners. This improvement suggested that the combination of SEL integration and careful attention to foundational concepts was supporting both confidence and competence development.

### 3.3. Cross-Case Synthesis: SEL Integration in Mathematics Education

The cross-case analysis revealed important patterns in how SEL integration manifested across different classroom contexts and implementation approaches. While all teachers were using the same JUMP Math curriculum, significant variations emerged in how SEL principles were enacted and the resulting impacts on learning environments and student outcomes.

#### 3.3.1. Teaching Energy and Educator Well-Being

Positive and supportive teaching energy was present in five of the six case study classrooms, fostered through steady instructional pacing, enthusiastic delivery, and appropriate use of humor. These qualities enabled students to focus more effectively on JUMP Math lessons while increasing overall engagement and learning. The responsive pacing allowed teachers to prioritize understanding and community building over rushing through content coverage, supporting both academic and social-emotional objectives. Even teachers with prior mathematics anxiety found that JUMP Math’s structured lessons provided the support and confidence they needed to deliver instruction with enthusiasm and enjoyment rather than stress and avoidance. The detailed lesson plans, clear progression of concepts, and embedded scaffolding reduced teacher anxiety while supporting their professional development in both mathematical content knowledge and pedagogical skills. Case 5 served as a crucial counterexample, illustrating how teacher stress, limited social-emotional competence, and poor implementation fidelity can undermine even well-designed curricula. The teacher’s strict adherence to behavioral control rather than the collaborative spirit of JUMP Math created an environment where behavioral management issues consumed instructional time and attention, making it difficult to establish the rhythm and community necessary for effective SEL integration. This contrast highlighted the vital importance of teacher social and emotional competence for successful implementation of SEL-integrated curricula. Teachers need not only training in curriculum content and procedures but also support for developing their own social-emotional skills and well-being.

#### 3.3.2. Learning Harmony and Social-Emotional Development

Across most JUMP Math classrooms observed, the structured inquiry approach fostered collaborative learning environments where classes progressed through lessons together, creating abundant opportunities for learning harmony and social-emotional development. The practice of moving forward as a learning community was evident to varying degrees in five of the six classrooms, with Case 5 showing significantly less evidence due to classroom management challenges and deviation from JUMP Math protocols. The other classrooms thrived through practices of progressing together, helping each other, and embracing mistakes as learning opportunities. These practices served multiple purposes: they created positive learning environments, empowered students as both learners and teachers, increased motivation and engagement in mathematics, and developed crucial social-emotional competencies including empathy, communication skills, collaborative problem-solving, and resilience. Students in these classrooms developed social awareness through recognizing peers’ different learning needs and approaches, relationship skills through collaborative problem-solving and peer support, and responsible decision-making through choosing how to contribute positively to the learning community. Some students became so engaged that they sought additional mathematical challenges outside of regular class time, suggesting that SEL integration supported rather than detracted from academic motivation and achievement.

#### 3.3.3. Emotional Stability and Psychological Safety

Five of the six case study mathematics learning environments demonstrated positive emotional stability characterized by respectful and responsive interactions, supportive feedback, and frequent invitations for participation rather than demands for compliance. This emotionally supportive environment was primarily established and maintained by teachers, with students mirroring the behaviors and interpersonal approaches modeled for them. The positive and emotionally stable learning environments appeared to support both effective mathematics teaching and learning and social-emotional development. Students felt safe to take intellectual risks, make mistakes, ask questions, and engage in challenging mathematical work when they experienced consistent support, encouragement, and respect from both teachers and peers.

Importantly, it was not the mathematical curriculum content itself that directly created these supportive environments, but rather the way that JUMP Math’s approach and philosophy were implemented by teachers who understood and embraced the SEL integration principles. The teacher’s role was crucial in translating curriculum design features into positive classroom experiences that supported both academic and social-emotional development. Case 5 provided a stark contrast, demonstrating how the absence of emotional stability and psychological safety significantly compromised both mathematics learning and social-emotional development. When the teacher did not establish supportive relationships, provide encouraging feedback, or create inclusive participation opportunities, both academic achievement and classroom community suffered, highlighting the essential connection between emotional climate and learning outcomes.

#### 3.3.4. Teacher Perspectives on SEL Integration Impact

The six case study teachers consistently endorsed JUMP Math and its approach to integrating SEL with mathematics instruction, even when implementation was challenging. Teachers found that the program helped their students become more successful mathematics learners while simultaneously boosting their own mathematical knowledge and confidence in teaching mathematics. Teachers appreciated that the lesson design scaffolded and supported both student learning and their own professional development as mathematics educators. The structured approach reduced their planning burden while increasing their understanding of mathematical concepts and effective pedagogical strategies. Several teachers expressed concerns about the quantity of material to cover and questions about whether students would be adequately prepared for subsequent grade levels. However, other teachers felt confident that their students were very well prepared for future mathematics learning, suggesting that variations in pacing and depth may reflect different implementation approaches or contextual factors rather than fundamental curriculum limitations. Teachers consistently observed improvements in student confidence, collaboration, persistence, and enjoyment of mathematics alongside academic achievement gains. They noted that students were more willing to help each other, take intellectual risks, persist through challenging problems, and view themselves as capable mathematics learners—all indicators of positive social-emotional development integrated with academic learning.

## 4. Discussion

This exploratory qualitative study employed a multiple case study approach to examine whether, and if so how, JUMP Math, a program explicitly designed with SEL principles, creates socially and emotionally supportive learning environments in Grade 5, 6, and 7 classrooms. Through lesson observations, teacher interviews, and check-in conversations, we investigated the qualities that characterize JUMP Math’s SEL-integrated mathematics instruction, how teachers and students experience this approach, and the role of implementation fidelity in creating supportive environments. From the lesson observations, three themes and related sub-themes emerged: (1) teaching energy: steady instructional pacing, enthusiastic delivery, infusions of humor; (2) learning harmony: progressing together, helping each other, embracing mistakes; and (3) emotional stability: supportive feedback, inviting participation, respectful and responsive interactions. These findings, in conjunction with insights from teacher interviews regarding their perspectives on SEL integration, math teaching, and students’ experiences, demonstrate how the instructional design and pedagogy of a mathematics curriculum can shape socially and emotionally supportive learning environments, while highlighting the integral role teachers play in enacting the curriculum with fidelity and responsiveness to learners’ social, emotional, and academic needs.

### 4.1. SEL Integration in Academic Instruction

This study provides evidence that mathematics curricula explicitly designed with SEL principles can create learning environments that simultaneously support academic achievement and social-emotional development, rather than requiring educators to choose between these important outcomes. The three themes—teaching energy, learning harmony, and emotional stability—reflect core SEL competencies being developed through mathematical activity rather than through separate, additional instruction. The findings align with contemporary calls for integrated approaches to SEL that leverage academic content as a vehicle for social and emotional learning (Schwartz et al., 2023) and calls that ignoring social and emotional needs compromise the very learning and academic achievement goals that teachers and schools are trying to achieve (Diamond, 2015, 2022, 2025). Rather than adding SEL as another subject competing for instructional time, JUMP Math demonstrates how mathematical instruction can inherently develop competencies in self-awareness (recognizing emotional responses to mathematical challenges), self-management (persisting through difficult problems), social awareness (recognizing peers’ learning needs), relationship skills (collaborative problem-solving), and responsible decision-making (choosing appropriate mathematical strategies and supporting classroom community). It also demonstrates the importance for mathematical learning outcomes of incorporating SEL into how math is taught. This integration appears particularly powerful because it occurs within authentic academic contexts where students are naturally encountering challenges, working with peers, making decisions, and developing competence. The social-emotional learning emerges from and supports academic learning rather than being artificially imposed or separated from meaningful content engagement.

### 4.2. Teacher Social and Emotional Competence

This study highlights the crucial role of teacher social and emotional competence in successful SEL integration, supporting Jennings and Greenberg’s (2009) Prosocial Classroom Model. Teachers who implemented JUMP Math with high fidelity demonstrated emotional awareness, social skills, and self-regulation that enabled them to create positive learning environments characterized by psychological safety, collaborative learning, and academic challenge. Several teachers reported that JUMP Math helped them overcome their own mathematics anxiety and develop greater confidence in mathematical instruction. This suggests that well-designed SEL-integrated curricula can support teacher professional development and well-being, creating positive cycles where confident, competent teachers are better able to implement programs effectively and support student development.

Conversely, Case 5 illustrated how teacher stress, limited social-emotional competence, and poor implementation fidelity can undermine even well-designed curricula. This teacher’s use of sarcasm, threats, and coercive management strategies created an environment where neither academic learning nor social-emotional development could flourish, despite access to the same curriculum materials and training opportunities as other teachers. Recent research on teacher use of sarcasm and verbal aggression suggests that these behaviors are predicted by teachers’ own social-emotional competence, personal well-being, and occupational health (S. S. Braun et al., 2024). Teachers experiencing stress, burnout, and limited social-emotional skills are more likely to engage in counterproductive interactions with students, highlighting the importance of supporting educator well-being and competence as a foundation for effective SEL integration (S. Jones & Ali, 2021).

### 4.3. Classroom Climate

In this multiple case study, the experience of JUMP Math lessons affected the overall social and emotional climate of the math-learning environment. The process of progressing together through the content, introducing students to multiple methods, embracing mistakes, and encouraging students to help each other were observed in all of the case study classrooms except the one with low fidelity to JUMP Math. These socially and emotionally supportive qualities of the math-learning environment evident during JUMP Math lessons (key aspects of the JUMP Math approach) helped create a positive classroom climate in which students were motivated and engaged in math learning and enjoyed positive interactions with each other and their teacher. It is well established that classroom climate affects students’ social, emotional, and academic outcomes (e.g., Pianta & Hamre, 2009). Indeed, López et al. (2023) found that classroom climate explained a significant portion of the variance in Grade 8 students’ math achievement and compensated for the negative effects of gender and socioeconomic status. Previous research has identified four qualities of classroom climate that support students’ math achievement: (a) autonomy support, (b) authentic instruction, (c) student collaboration, and d) teacher social support (Patrick et al., 2011; Wigfield et al., 2006). All four of these qualities were evident during JUMP Math lessons in the five case study classrooms with high fidelity to the approach. Autonomy support was encouraged through giving students choices about which strategies to use and inviting their input into how to solve a problem. Authentic instruction was evident in how teachers made connections from lesson topics to students’ lives and the practical applications of math beyond simple memorization of algorithms. Student collaboration was highlighted in most case study profiles under the sub-theme *helping each other.* Finally, teacher social support was evident (described in the Section 3 sub-theme *respectful and responsive interactions*). That these qualities were apparent during most JUMP Math lessons observed is not an accident; they are integral aspects of the program.

### 4.4. Implications for 21st Century Education

This study’s findings have several important implications for educational practice, particularly as educators seek approaches that prepare students for contemporary challenges while also addressing their academic and social-emotional needs.

#### 4.4.1. Curriculum Design

Mathematics curricula can be designed to inherently support SEL development without sacrificing academic rigor or achievement. Key design principles emerging from this study include: (a) *Collaborative learning structures* that provide regular opportunities for peer interaction, support, and shared problem-solving; (b) *Mistake normalization practices* that frame errors as learning opportunities and reduce anxiety about mathematical challenges; (c) *Scaffolded challenge* that maintains students in productive struggle zones where they experience appropriate difficulty with adequate support; (d) *Teacher support materials* that build educator confidence and competence in both mathematical content and pedagogical approaches; and (e) *Flexible pacing* that prioritizes understanding and community building over rigid adherence to timelines. These design features can be incorporated into various mathematical curricula and instructional approaches, suggesting broader applicability beyond the specific JUMP Math program examined in this study.

#### 4.4.2. Teacher Professional Development

Professional development for mathematics educators should address both mathematical content knowledge and social-emotional competence, recognizing that effective SEL integration requires teachers skilled in both domains. Key components should include: (a) *Mathematical content knowledge* development to build teacher confidence and reduce mathematics anxiety; (b) *Social-emotional learning competencies* to support teachers’ own well-being and their ability to create positive learning environments; (c) *Implementation support* including modeling, coaching, and ongoing feedback to ensure high-fidelity curriculum implementation; (d) *Collaborative learning facilitation skills* to help teachers create classroom communities that support both academic and social-emotional development; and, (e) *Stress management and self-care strategies* to support teacher well-being and prevent burnout that can undermine effective practice.

#### 4.4.3. Assessment and Evaluation

Evaluation of mathematics programs might consider social and emotional outcomes alongside academic achievement, recognizing that these domains are interconnected and mutually supportive. The current study suggests that SEL integration may contribute to academic success through improved classroom climate, increased student engagement, and enhanced motivation to persist through mathematical challenges. Assessment approaches might include measures of: (a) *Student social-emotional competencies* including collaboration skills, resilience, and mathematical confidence; (b) *Classroom climate* characteristics such as psychological safety, peer support, and positive relationships; (c) *Teacher well-being and competence* including confidence in mathematical instruction and social-emotional skills; and (d) *Long-term outcomes* such as sustained interest in mathematics and transfer of social-emotional skills to other contexts.

#### 4.4.4. Connections to Contemporary Educational Priorities

The present findings connect to several contemporary educational priorities and challenges facing 21st-century schools. For example, SEL-integrated mathematics instruction appears to create more inclusive learning environments where diverse learners can succeed. The emphasis on collaboration rather than competition, multiple approaches to problem-solving, and support for different learning needs aligns with equity goals in mathematics education. Students who might struggle in more traditional, individualistic approaches find success and develop positive mathematical identities in these collaborative, supportive environments. Students who lacked confidence in their ability to learn and succeed at mathematics discover mathematics is not so difficult after all and they can succeed at it (Mighton, 2003). Importantly, JUMP Math emphasizes leaving no student behind. Mathematics instruction at the beginning of the school year starts at the level of the student who is most behind. Most math curricula do not do that, and thus the students most behind cannot follow the lessons, leading to their being frustrated, feeling “stupid” and depressed, and often disrupting everyone else’s learning—with the result that other mathematics curricula produce less learning gains by the end of the year than does JUMP Math (Murray, 2019; Solomon et al., 2019).

The present study also provides evidence that academic instruction can contribute to student and teacher well-being rather than creating additional stress or anxiety. Mathematics, often a source of anxiety and negative emotions, became a context for building confidence, resilience, and positive relationships when delivered through an SEL-integrated approach. The collaborative problem-solving, communication skills, and persistence developed through SEL-integrated mathematics instruction prepare students for the complex, interdisciplinary challenges they will face in future educational and professional contexts (Yoder et al., 2020).

### 4.5. Theoretical Contributions

The present findings contribute to the theoretical understanding of how SEL integration occurs within academic contexts and the conditions that support successful implementation. The three themes that emerged here provide a framework for understanding the multifaceted nature of SEL integration, encompassing teacher behaviors (Teaching Energy), peer interactions (Learning Harmony), and classroom climate (Emotional Stability). Further, the findings support and extend the Prosocial Classroom Model (Jennings & Greenberg, 2009) by demonstrating how curriculum design can support teacher social-emotional competence and classroom climate, creating positive feedback loops between teacher development and student outcomes. They also further our understanding of how growth mindset principles can be embedded in curriculum design and instructional practices rather than simply taught as abstract concepts.

### 4.6. Limitations and Future Research

This study has limitations that should be considered when interpreting its findings. For example, its qualitative and exploratory design and small sample size limit the generalizability of findings. However, this approach did allow for an in-depth examination of classroom dynamics and valuable insights into how SEL integration manifests in authentic classroom contexts. Another limitation is that data collection, coding, and analysis were led by a single researcher. Although analytic rigor was supported through peer debriefing, triangulation of multiple data sources, and the maintenance of a detailed audit trail, future studies should incorporate multiple coders and interrater reliability checks to further strengthen the trustworthiness of the findings. A third limitation is that the study did not include student interviews or direct measures of student outcomes. Incorporating student perspectives and directly measuring academic and social-emotional outcomes represent important next steps for future investigations. A fourth limitation is that the study is necessarily limited to communities it studied. Replication studies in different communities, with students from different backgrounds, and with students with special needs will be important to undertake. A final limitation is that all participating teachers were white females, while four of the six classrooms served student populations that were majority students of color. While we observed successful SEL integration across these classrooms, the dynamics of creating emotionally supportive environments may manifest differently with teachers of different genders, races, or cultural backgrounds. The cultural congruence between teachers and students can influence relationship-building, communication patterns, and the interpretation of social-emotional cues.

Several directions for future research would strengthen our understanding of SEL integration in mathematics education and better understanding of JUMP Math specifically. For example, future studies should employ mixed-methods approaches with larger sample sizes, control groups, and quantitative measures of SEL competencies, classroom climate, and academic achievement. This would enable more definitive conclusions about the effectiveness of SEL integration and identification of specific factors that contribute to positive outcomes. Longitudinal studies could examine whether students who experience SEL-integrated mathematics instruction develop stronger social-emotional competencies and stronger mathematical competencies over time and whether these benefits transfer to other subjects and life contexts. Such research could also investigate the sustainability of benefits and optimal duration of exposure to SEL-integrated approaches. Future research should also explore specific implementation factors that support successful SEL integration, including teacher preparation programs, professional development models, administrative support, and organizational conditions. Understanding what enables some teachers to implement with high fidelity while others struggle could inform more effective support systems. Studies in more diverse educational contexts, including different grade levels, cultural settings, and socioeconomic environments, would help elucidate the generalizability and cultural responsiveness of SEL integration approaches. Research should also examine how SEL integration might need to be adapted for different populations and contexts. Finally, future research could examine how specific SEL competencies develop through mathematics instruction and which curriculum features most effectively support different aspects of social-emotional learning. This research could be grounded in a framework based on the dimensions of teaching energy, learning harmony, and emotional stability identified in this study.

## 5. Conclusions

This multiple case study demonstrates (a) that mathematics education can serve as a powerful context for social and emotional learning (SEL) and (b) addressing social and emotional needs can contribute to learning goals for the academic content, when curricula are explicitly designed with SEL principles and implemented by teachers who understand and embrace these approaches. The JUMP Math program illustrates how academic instruction can simultaneously develop mathematical competence and social-emotional skills through collaborative learning, mistake normalization, and supportive classroom climates. The findings contribute to understanding how SEL can be integrated into academic instruction to benefit both student well-being and achievement, addressing contemporary calls for educational approaches that prepare students for 21st-century challenges while supporting their holistic development. The three themes—teaching energy, learning harmony, and emotional stability—provide a framework for understanding the multifaceted nature of successful SEL integration in math-learning environments. The teacher’s role remains crucial in this integration, highlighting the need for approaches that support educator well-being and competence alongside student development. When teachers feel confident and supported in their mathematical instruction, they are better positioned to create the emotionally supportive environments that enable both academic learning and social-emotional growth. As educators continue seeking approaches that address both academic and social-emotional needs efficiently and effectively, curricula that integrate these domains, like JUMP Math, offer promising possibilities for creating more supportive, inclusive, and successful learning environments. The evidence from this study suggests that such integration not only is possible but seems to enhance, rather than compromise, academic achievement while developing social-emotional competencies essential for success in school and in life. Importantly, the present findings suggest a potential mechanism contributing to the superior academic outcomes that others have reported for JUMP Math students (Murray, 2019; Solomon et al., 2019). Our qualitative evidence indicates that attention to social-emotional needs—through collaborative structures, mistake normalization, and emotionally stable environments—may enhance students’ willingness and capacity to engage with and master mathematical content, thereby contributing to the academic gains observed in quantitative studies. These findings suggest that addressing social-emotional needs may be a key factor enabling JUMP Math to achieve outcomes that surpass other mathematics curricula. Future research and practice should continue to explore (a) how academic subjects can serve as vehicles for developing the social and emotional competencies that students need to thrive in an increasingly complex, interconnected world and (b) how addressing social and emotional needs within the context of academic instruction can serve to enhance student gains from the academic instruction.

## Figures and Tables

**Table 1:** Case Study Demographic Information (Teacher and Classroom).

Case ID	Teacher Gender	Teacher Ethnicity	Years Teaching	Years Teaching JUMP Math	Grade Level	Classroom Composition (Students)		
						*n*	Gender	Ethnicity
School A								
Case 1	Female	White	20	15	5	28	~50% F	>50% white
Case 2	Female	White	14	8	6	25	~50% F	>50% white
School B								
Case 3	Female	White	19	2	7	21	<50% F	>50% POC
Case 4	Female	White	22	1	5	27	>50% F	>50% POC
Case 5	Female	White	8	2	5	26	~50% F	>50% POC
Case 6	Female	White	13	1	5	25	~50% F	>50% POC

**Table 2:** Qualities of the SEL-Integrated JUMP Math Learning Environment.

Themes	Sub-Themes	Examples
Teaching energy	Steady instructional pace	Focused, evenly paced instruction keeping learners engaged and encouraging active participation
	Enthusiastic delivery	Dynamic body movements, excited tone of voice, and open facial expressions during lesson delivery
	Infusions of humor	Making light of computational or other mistakes, and other light-hearted expressions of humor, to introduce levity and fun into the learning environment
Learning harmony	Progressing together	Students answering questions out loud and in unison, and moving at the same pace through the content
	Helping each other	Students encouraging and assisting each other to develop their thinking, expand their explanations, and use mathematical language
	Embracing mistakes	Teachers and students viewing mistakes as opportunities to learn, taking time to explore the mistake and correct it together
Emotional stability	Supportive feedback	Comfort and confidence in math learning encouraged through frequent, positive feedback
	Inviting participation	Teachers and students inviting students to participate and share their thinking and ideas
	Respectful and responsive interactions	Teachers and students using perspective-taking skills to understand others and respond according to their social and emotional needs

## Data Availability

The data presented in this study are available upon request from the corresponding author due to privacy reasons.

## Abbreviations

The following abbreviations are used in this manuscript:

SEL	Social and Emotional Learning
POC	People of Color
